# Comparison of Adenosine Deaminase, C-reactive Protein, Uric Acid, and Rheumatoid Arthritis Levels in Patients With Rheumatoid Arthritis and Those Without Arthritis: A Review

**DOI:** 10.7759/cureus.57433

**Published:** 2024-04-01

**Authors:** Priyanka A Makhe, Anjali A Vagga

**Affiliations:** 1 Biochemistry, Jawaharlal Nehru Medical College, Datta Meghe Institute of Higher Education and Research, Wardha, IND

**Keywords:** healthy control, non-arthritis patients, rheumatoid arthritis, c-reactive protine, ra factor, adenosine deaminases, uric acid

## Abstract

One of the hallmarks of rheumatoid arthritis (RA) is inflammation of the synovial membrane, and oxidative stress is a mediator of tissue damage. RA is characterized by persistent joint inflammation, which leads to pain, edema, and finally joint destruction. Numerous biochemical markers can cause RA because of their impact on systemic and local inflammation. Numerous biomarkers have been investigated for their potential application in the diagnosis and prognosis of RA. In this review article, we evaluate the role of RA factor or rheumatoid factor (RF), uric acid, C-reactive protein (CRP), and adenosine deaminases (ADAs) as biomarkers in patients with and without arthritis. Studies that analyze and compare the levels of uric acid, ADAs, CRP, and RF in patients with and without arthritis. Although recent research has shown higher levels of uric acid, ADA, CRP, and RA in patients with RF compared to healthy controls, these findings may indicate a role for these markers in reflecting inflammation and disease activity. In the metabolism of purines, the enzyme ADA is involved. The liver produces CRP, which is then released into the bloodstream. In inflammatory situations, there is a rise in CRP levels. This biomarker is frequently used for systemic inflammatory assessment in RA. The pathophysiology and severity of RA have both been connected to uric acid, which has historically been linked to gout. One particular biomarker for RA is RF. When compared to a healthy control group of individuals with arthritis, this review provides valuable insights into the diagnostic and prognostic use of uric acid, CRP, ADAs, and RF.

## Introduction and background

Rheumatoid arthritis (RA) is a persistent systemic inflammatory illness characterized by joint erosions, abnormalities, and inflammation. RA affects 1% of persons worldwide, with a 3:1 incidence rate in women compared to men. In India, RA affects about 10 million people. Women are more susceptible to RA than men, particularly those between the ages of 40 and 60. In addition, compared to those of the same age and gender without the illness, those with RA have a 1.5 to 2 times higher risk of developing cardiovascular disease (CVD) [1]. RA, the most prevalent systemic autoimmune disease, results in functional impairment and early mortality. Roughly 70% of patients experience irreparable joint degeneration and 80% of young adults in the workforce who are actively involved with RA experience excruciating pain and stiffness. This leads to a major loss of everyday activities and professional productivity, which significantly lowers quality of life [2].

It has been demonstrated that biomarkers for RA, such as RA factor or rheumatoid factor (RF) and anti-cyclic citrullinated peptide (anti-CCP), exist before the clinical diagnosis of RA. The anti-CCP antibody is particularly predictive of the development of RA in the future [3]. Early diagnosis methods for RA have significantly improved after the discovery of anti-citrullinated protein antibodies and anti-carbamylated protein antibodies. RFs were discovered eight decades ago. In patients with RA, smoking has also been linked to a higher prevalence of RF [4]. In 1948, Rose was the first to identify serum gamma-globulins as antibodies in patients with RA. Due to their correlation with RA, they were dubbed *RFs* in 1952 [5]. Adenosine deaminase (ADA) is responsible for breaking down adenosine. As a crucial component of cell-mediated immunity and an enzyme involved in purine metabolism, ADA induces inflammation and has been related to several infectious and inflammatory diseases. Cell-mediated immunity is also represented by ADA, which indicates the maturation and activity of lymphocytes as well as the differentiation of monocytes into macrophages. There is a connection between RA and elevated ADA levels in serum and synovial fluid [6]. ADA activity is usually measured in the range of 14-22 mmol/L [7].

Furthermore, in cases of Behcet's illness, juvenile idiopathic arthritis, and systemic lupus erythematosus (SLE), ADA has been proposed as an alternate measure of disease activity [8]. ADA in synovial fluid is also capable of distinguishing between noninflammatory and RA. Determining the adenosine level in serum and synovial fluid can, therefore, be a useful method of evaluating disease activity [9]. Elevations of ADA activity in synovial fluid might indicate the level of joint inflammation and are closely correlated with the activity of underlying diseases. Consequently, ADA as a marker of cellular immune activation, may aid in the relief of inflammation-triggering elements, the advancement of novel therapeutic strategies, and a better knowledge of certain pathophysiologic features of the disease [10]. In artery walls and red blood cells, irreversible hydrolytic deamination of adenosine to inosine and 2'-deoxyadenosine to 2'-deoxyinosine occurs due to the enzyme ADA. This process yields xanthine, hypoxanthine, and ultimately uric acid (UA) from inosine and 2'-deoxyinosine. Based on the strong correlation between serum UA and levels of interleukin-6 (IL-6), C-reactive protein (CRP), and tumor necrosis factor (TNF) in the blood, inflammatory diseases like RA may be associated with systemic inflammation. Patients with RA may benefit from testing their serum levels of ADA and UA to ascertain their oxidant-antioxidant status [11]. Since UA has a plasma concentration 10 times higher than that of vitamins C and E, it has long been thought to play a beneficial role as an endogenous antioxidant. UA is a widespread by-product of purine metabolism. It contributes two-thirds of plasma's ability to scavenge free radicals [12]. RF can be present in people with a range of non-rheumatic arthritic conditions. Normally, CD5-positive B cells produce polyreactive, low-affinity IgM antibodies on their own as an RF [13]. Serum UA has been pathogenically associated with hypertension (HT) and CVD. The development of CVD in general and possibly in the population with RA is predicted by serum UA, which is an independent, significant factor that roughly doubles the frequency of HT [14].

RFs can be detected in both healthy individuals and patients with different autoimmune and non-autoimmune disorders. In other illnesses, the RF is also present. Certain connective tissue illnesses, such as RA, may be associated with other conditions, including primary Sjogren's syndrome and SLE. Moreover, people with specific illnesses, including hepatitis C, rubella, malaria, and post-vaccination, may have higher RF levels [15]. Degradation of the synovium and inflammation are common causes of slow joint degeneration. Worldwide, between 1% and 2% of people are affected by RA. Approximately 70% of people have an irreparable joint injury and 80% of young adults with jobs that require physical activity report having significant joint pain and stiffness. Between 0.5% and 1% of individuals worldwide are affected by RA [16]. According to reports, RF testing in individuals with RA has a sensitivity of 60% to 90% and a specificity of 85%. However, the range of sensitivity could be anywhere from 26% to 90%, depending on the selected patient and control population [17]. Researchers discovered a repertoire of B-cells with the ability to release RF (RF + B-cells) in the peripheral blood of healthy individuals. RF synthetization requires a particular activation pattern, and this population appears to be anergic in people who are RF- seronegative [18]. CRP is one of the acute phase serum reactants that the liver responds to the highest. CRP, which is generated by a range of pro-inflammatory cytokines that are either derived from monocytes or macrophages, is a marker of more variable fluctuations in disease activity related to joint degeneration. CRP levels of patients with RA were notably greater than those of controls. Studies on CRP levels in synovial fluid and serum from patients with RA have revealed a strong correlation between CRP and IL-6 levels [19]. The level of CRP is commonly used to measure the degree of disease activity in RA, in conjunction with assessments of articular swelling and discomfort [20].

## Review

Rheumatoid arthritis

The word *disease of the joints* in Greek is the origin of the phrase arthritis. It is described as either a sudden or persistent inflammation of the joints, frequently accompanied by discomfort and structural damage. Arthroralgia, which describes pain that is restricted to a joint, regardless of the source of the pain, is not the same as arthritis. RA is a form of autoimmune systemic inflammatory illness. Although family history is a significant risk factor, it is unclear if this indicates a shared environmental exposure or a genetic vulnerability [21]. The immune system becomes activated and dysregulated due to a combination of environmental factors, such as smoking, and various genetic factors, including HLADRB1 among others, leading to inflammation in RA. A recent study also discovered that smoking is a substantial risk factor for immune-mediated disorders, such as the common HLA-DR epitope, which is linked to an elevated risk of RA factor (RF)-seropositive RA [22]. RA can manifest clinically in a variety of ways, but the most typical observation is a slow onset of pain that is accompanied by symmetric swelling of the tiny joints. Only around 25% of people develop acute or subacute RA; other probable presentation patterns include palindromic, monoarticular, extra-articular synovitis, polymyalgia-like symptoms, and nonspecific symptoms such as fatigue, fever, malaise, and weight loss. The fact that RA is a symmetric arthritis is one of its characteristics [23]. Articular and periarticular symptoms include, for example, joint swelling and tactile irritation. Extreme impairment of mobility and stiffness in the mornings may also be present in the affected joints [24].

RA factor

The RF antibody family, which comes in a variety of isotypes and affinities, targets the Fc region of immunoglobulin G (IgG). Although IgG and IgA are also infrequently mentioned, IgM is the most commonly stated RF. Most likely, the evolution of human RFs served as a means of assisting in the elimination of immune complexes from the circulatory system [25]. Numerous additional pathologies, such as RA, non-autoimmune diseases, and autoimmune disorders are associated with RFs. They have been found in as many as 4% of young and aged, healthy people. Although some individuals with early RA may develop RF later in the course of the disease, between 30% and 45% of patients do not have RF [26]. The majority of B cells in normal human lymphoid tissue express RF on their cell surface. However, in the absence of an antigenic trigger, RF is not consistently detected in the bloodstream. Modified IgG, the release of heavy chains from B cells that have undergone apoptosis, and elements of microbial organisms, like the Epstein-Barr virus may act as catalysts for the creation of RF and may play a significant role in the pathophysiology of RA. RF typically ranges from 0 to 20 IU/mL. RF levels beyond 20 IU/mL are thought to be insufficient for diagnosing RA because they can be increased for other reasons [27].

*Relation Between RF* *in Patients With Arthritis and Healthy Controls*

It has been demonstrated that low-affinity RFs play a significant role in immune responses to various infections, while RFs with strong affinity are linked to more intense and protracted disease among individuals with RA. RF is a significant predictor of a bad outcome in people with RA as compared to healthy controls. Two high titers predict more impairment, extra-articular symptoms, and higher joint degradation [28]. Rheumatoid nodules are caused by RF that has been accumulated in tissue. Approximately 70% of patients with RA have positive RF tests at the outset of the disease, and 85% test positive during the first two years of the illness. Since serum RF levels fluctuate slowly, it is impossible to track the progression of an illness using them [29].

Adenosine deaminase

The hydrolytic deamination of adenosine to inosine and deoxyadenosine to deoxyinosine is catalyzed by the enzyme ADA, which is involved in the metabolism of purines. This enzyme regulates how these metabolites affect vascular, neurological, and immune systems in a physiologically significant way [30]. ADA is crucial for the development and multiplication of lymphocytes, especially T-cells. ADA activity is elevated in disorders such as typhoid fever, infectious mononucleosis, liver disease, sarcoidosis, leukemia, brucellosis, acute pneumonia, RA, malignancies, and tuberculosis that are linked to cellular system activation [31]. The enzyme ADA is essential for the digestion of adenosine generated from food and for the metabolism of nucleic acids in tissues, but its main function at the moment is the growth and maintenance of the immune system because it is crucial to lymphocyte development and function. Adenosine receptors are members of the G protein-coupled receptor family. There are four subtypes of adenosine receptors (A1, A2A, A2B, and A3), and each one has a distinct function in controlling proper cellular physiology in a range of tissues, such as the brain, heart, and lungs [32]. ADA type 2 (ADA2), the predominant form seen in plasma, is elevated in several illnesses, especially immune-related ones like RA. The fact that ADA2 and ADA1 coexist only in monocytes and macrophages suggests that ADA2 is not widely distributed [33].

Relation Between ADA in Patients With Arthritis and Healthy Controls

Serum ADA levels were shown to be greater in patients with RA compared to healthy controls, suggesting the usefulness of this marker in the identification of the illness. Extracellular ADA is created during RA inflammations, and this leads to a significant increase in cell-mediated immune activity. It suggests that this could be evaluated as a diagnostic marker for a patient with RA. Blood levels of ADA may be able to predict disease activity in patients with RA [34]. ADA activation may be a useful biochemical marker of the inflammatory process in individuals with RA since it is intimately linked to inflammation. To distinguish between distinct infectious and malignant diseases in bodily fluids, serum ADA is frequently utilized. To explain changes in immunity, which show changes in monocyte/macrophage turnover or activity, which is responsible for the higher levels of ADA in serum reported in individuals with RA. Serum ADA levels were higher in patients with RA, according to several studies [35].

Uric acid

Purine degradation results in UA. Ionized urate, or UA, circulates at the normal physiological pH of 7.4. Purine metabolism can happen in any tissue that has xanthine oxidase, including cardiac and pulmonary tissue, but it mostly happens in the liver. Roughly two-thirds of the UA that the body produces is eliminated by the kidneys, with one-third entering the intestine. Ninety percent of the urea filtered and excreted by the kidneys is reabsorbed in the proximal tubule [36]. The urine usually gets rid of UA. High blood UA levels can cause UA crystals to develop and precipitate in the joints, which can lead to gouty arthritis. Elevated UA may affect RA autoimmunity in an animal model, as per a study by Turner in 1983. The condition known as hyperuricemia is characterized by elevated serum UA values greater than 7 mg/dL. This disease causes gout and quickens the course of renal and cardiovascular diseases. UA can build up in the blood and tissues due to a metabolic disease called gout [37].

*Relation Between UA*​​​​​​* in Patients With Arthritis ​​​​​​​and Healthy Controls*

UA is a protective antioxidant that effectively neutralizes radicals such as superoxide, hydroxyl, and peroxynitrite, thereby preventing lipid peroxidation. However, when contrasting the UA concentrations of individuals with RA to those of healthy individuals, there isn't a discernible difference. Patients with RA may have elevated levels of ADA, which could explain the increase. UA is best known for its role in gout. When UA concentrations exceed the limit of solubility, crystal formation can ensue, which is capable of activating the NALP3 inflammasome and triggering the acute severe attacks of joint inflammation characteristic of gout [38].

C-reactive protein

CRP belongs to the pentraxin protein family, which includes innate immune response-related pattern recognition molecules. It is an acute-phase reactant. CRP can be found in both infectious and noninfectious conditions, and it is elevated in both acute and long-term inflammatory conditions. Tillett and Francis discovered the CRP in 1930 [39]. The material in the serum of patients experiencing acute inflammation was originally found to react with the *C* carbohydrate antigen of the pneumococcus capsule, hence the name CRP. The liver produces the pentameric protein, that is, CRP. Its concentration increases as inflammatory processes occur [40]. Acute-phase reactant protein, or CRP, is primarily generated by the action of IL-6 on the gene that codes for CRP transcription during the early phases of an infectious or inflammatory event. Using attachment sites such as phospholipids, chromatin, phosphocholine, and histones makes the identification and removal of invading pathogens and damaged cells easier by utilizing fibronectin [41].

*Relation Between CRP in Patients With Arthritis​​​​​​​ and Healthy Controls*

Patients with RA have higher levels of CRP, a nonspecific measure of systemic inflammation. Inflammation and CRP are connected. Before the development of RA, serum samples from patients with RA showed a higher frequency of elevated CRP concentrations, according to certain studies. Patients with RA have high levels of CRP, a sensitive indicator of systemic inflammation. RA is one type of inflammation that can be brought on by elevated levels of CRP in patients with arthritis as compared to controls [42].

Serum ADA, CRP, and RF levels were shown to be considerably higher in patients with RA when compared to patients without RA in a study by Garg et al. involving 102 people in total, 52 of whom were patients with RA and 50 of whom were patients without RA. Regarding UA levels, there was no significant difference (*P *< 0.02) between patients with RA and those without RA [43].

## Conclusions

In conclusion, the evaluation of ADA, CRP, UA, and RF levels offers significant perspectives on distinguishing between individuals with and without arthritis. Discovering potential biomarkers linked to non-arthritic diseases and RA was the goal of the study. Increased levels of serum ADA have been noted in people with RA, suggesting that this test could help diagnose the illness. During inflammation caused by RA, extracellular ADA is produced, which significantly boosts cell-mediated immunity. Accordingly, ADA may function as a biomarker to differentiate between cases of RA and non-RA. The chance of developing symptoms of arthritis is increased by CRP, which is elevated by all infections. Patients with RA had considerably higher serum CRP levels. Levels of UA are frequently linked to gout; however, they do not provide any useful information in cases of RA. UA levels in patients with RA strongly predict renal impairment. Patients with increased UA who have RA may need to be screened for renal failure and given the proper medication. As a differential diagnosis, measuring UA levels in addition to other indicators can still help rule out gout. Immunoglobulin is the RF. RF levels rise in patients with arthritis. Anti-CCP antibodies and RF are two specific biomarkers linked to patients with RA. When compared to non-RA, elevated levels of these biomarkers in patients with arthritis validate the existence of autoimmune activity typical of RA.
